# Editorial: Governing sustainability transitions in agribusinesses and food-systems: a behavioral foundations view

**DOI:** 10.3389/fpubh.2023.1176045

**Published:** 2023-08-14

**Authors:** Maria Carmela Annosi, Lucia Marchegiani, Esteban R. Brenes

**Affiliations:** ^1^School of Social Sciences, Wageningen University and Research, Wageningen, Netherlands; ^2^Department of Business Studies, Roma Tre University, Rome, Italy; ^3^INCAE Business School, Alajuela, Costa Rica

**Keywords:** behavioral view, sustainability transition, innovation diffusion and acceptance, decision-making mechanisms, inclusion in agriculture, lone-working, agribusiness

Today, we face fundamental sustainability challenges in several domains such as agriculture and food systems, which are affected by strong inter-dependencies. Against this backdrop, the question of how to encourage sustainable modes of production and consumption is garnering more attention in the policy arena and upholds the need for empirical and conceptual research in sustainability transitions. Sustainability and sustainable development are multi-faceted concepts, and a holistic approach to sustainability demands respect for environmental, economic, and social issues. Coherently, the articles in this Research Topic tackle the challenges of sustainability transitions from different angles and show interesting arguments for further developing a research agenda on sustainability transition in agribusiness and food systems, shedding more light on: environmentally sustainable modes of production; social sustainability and decent work in agribusiness organizations; socio-economically sustainable dynamics of innovation acceptance and diffusion; socially sustainable policies to promote a culture of holistic sustainability and new values; sustainability of digitalization and role of digital platforms in driving producers' wellbeing and consumers' behaviors.

Many forces act on farmers and shape their behaviors toward the realization of more sustainability principles. Among them, the study of Teng et al. identifies the relevance of individual characteristics, government guiding factors, industrial organization-promoting factors, and market adjustment factors. These factors highlight the complexity of shaping behaviors across the transition toward sustainability and consider behavioral changes as a result of social processes favoring the dissemination and assimilation of new values, which will shape the rules of conduct of guiding actors through induced multilevel interactions and interventions from relevant actors. In addition, research shows that farmer green production behavior has a positive impact on the quality and safety of agricultural production. However, if the first article has examined the structure in which farmer green production behavior occurs, the article by Dong et al. investigates the degree to which differences in individual characteristics can explain the different mobilization of resources activating sustainable entrepreneurship behavior. This second article shows that in increasing their self-efficacy and providing them with a sense of their ability, farmers experience higher entrepreneurial activities. Along a similar line of reasoning, the article by Lu et al. shows that a combination of cognitive levels, such as individual power, positively affects the behavioral decision-making mechanisms of landless farmers. Additionally, following a multilevel approach, the authors argue that the individual cognition of farmers is influenced by a combination of individual, territorial, and cultural power (1). In line with this, Ren has shown how the presence of a local agricultural socialized service (i.e., affecting the perception of territorial and cultural power) has a significant positive impact on farmers' organic fertilizer application behavior and can effectively alleviate the inhibitory effect of risk perception on this behavior. However, power also increases feelings of self-sufficiency and decreases the willingness to help others (2) and the wish to contact others (3). Indeed, power decreases people's consideration of others' perspectives (4) and their compassion for others' suffering (5). In addition, power increases dehumanization (6) and heightens the objectification of others, whereby people treat others to further their own goals (7). These behaviors do not represent the actions of a socially engaged individual. In the case of farmers, past studies, despite largely not being focused on feelings of loneliness and isolation within farming, have recognized that loneliness characterizes a variety of international farming contexts. For instance, Lunner Kolstrup et al. (8) report a reduction of social interactions between family members and neighbors of Swedish dairy farmers and feelings of isolation among migrant agricultural workers caused by cultural and linguistic barriers. Similarly identify lone working and geographical isolation as limiting farmers' opportunities for wider social interaction in Wales. As reported in the article by Kallionemi et al., loneliness is one of the major factors affecting farmers' mental health, even more so than physical workload. Furey et al. (9) identify lone working and restricted access to social support among farmers in Ireland as contributors to stress. There is a need to ease loneliness in order to avoid the serious consequences for the sustainability of farming, among others, by lowered work ability, health problems, and sleep difficulties. This raises the issue of how to make the field of agriculture more inclusive, paying attention to small and remote farmers, as suggested in the article by Mohapatra et al.. They argue that conditions should be created for farmers to incentivize the adoption of and access to technology and digital tools such as climate-smart financial innovations and better information, machinery, and equipment, as well as the possibility of learning from other producers through social private and public organizations. This would help them reach the target of a sustainable and inclusive agribusiness model, thus successfully facing their vulnerability and climate change.

The use of the internet and the consequent establishment of online connections can indeed affect the status of loneliness and social support (10). Individuals can be more prone to sharing personal information than when they have face-to-face interaction, helping to establish strong ties with others. Additionally, people also have the conditions to more easily express and experiment with some aspects of their identities. Accordingly, many studies have expressed the positive effects of internet usage and online connections on mental health (10). The article by Yang et al. underlines the social support that online communications offer farmers, leading them to decide to adopt a new technology. Currents studies have strongly emphasized the role of structural support that farmers receive as an enabler of technological adoption (11) during the technological transition, including the role of intermediaries [see the role of agricultural contractors in Kutter et al. (12)], or for the role of third parties, see Klerkx and Leeuwis (13) or the role of social networks [see the organic network of Kroma (14)]. However, the role of digital platforms as a source of social support, helping to reduce the status of loneliness and isolation for farmers and providing the means to face the complexities of the market, has been neglected. Indeed, digital platforms have been analyzed as an intermediation function (15, 16) or vehicle to coordinate multiple stakeholders (17) or as a tool to help diffuse new practices (18), but they have not been analyzed for their impact on individual mental status. Digital platforms provide opportunities for better user experiences through the possibility to develop an omni-channel environment, as shown in the article by Liu and Zheng. They provide agriculture retailers with a better understanding of omni-channel user experience, showing how this can affect their social and economic sustainability by warning about the mechanisms of social influence (19) and confirming the role of positive online reviews and reference groups.

## Author contributions

All authors listed have made a substantial, direct, and intellectual contribution to the work and approved it for publication.
